# Silver oxide decomposition mediated direct bonding of silicon-based materials

**DOI:** 10.1038/s41598-018-28788-x

**Published:** 2018-07-11

**Authors:** Tomoki Matsuda, Kota Inami, Keita Motoyama, Tomokazu Sano, Akio Hirose

**Affiliations:** Division of Materials and Manufacturing Science, Graduate School of Engineering, Osaka University, 2-1 Yamadaoka, Suita, Osaka, 565-0871 Japan

## Abstract

Silicon-based materials are widely promising electronic components by the combination with metals in power electronics field. However, bonding metal and silicon-based materials generally requires specific surface modification due to their different chemical bonds. Here, we demonstrate a process for directly bonding metals to silicon-based materials that does not require surface treatment, based on the *in situ* decomposition of Ag_2_O paste, forming Ag nanoparticles (AgNPs). We demonstrate sound joints of Ag/silicon-based materials at 300–500 °C with the formation of a silicon oxide interlayer containing AgNPs. We propose that Ag in the interlayer attracted other Ag particles to the interface, playing a unique role in this direct bonding process. This process is suitable for various bonding applications in electronics, as well the fabrication of conducting paths for photovoltaic and other applications.

## Introduction

The bonding between metal and silicon-based materials (Si-material), such as silicon carbide (SiC), a wide-bandgap semiconductor, and silicon dioxide (SiO_2_), used as gate oxide in metal-oxide-semiconductor (MOS) structures, has attracted growing interest in the recent development of the power electronics field^1^. Since covalent and ionic bonds are generally the principal types of chemical bonds in these semiconductors, compatibility with the metallic bonds is greatly limited. Hence, some surface pre-treatments, e.g. metallization, are necessary to facilitate bonding between metal and semiconductors (or various ceramics)^2–5^. For example, titanium or nickel have been deposited on Si^2,3^ and SiC^4,5^ substrates. Hence, conventional metal-to-ceramic bonding involves indirect processes.

A bonding method involving the sintering of Ag derived from the redox reaction between Ag_2_O and an organic solvent has been reported^6,7^: Ag nanoparticles (AgNPs) are generated during the bonding process as a result of the redox reaction. Nanoparticles smaller than 10 nm show an apparent melting-point depression^8–10^ caused by the high surface to volume ratio^11^, making them more reactive. Recent studies reported that, not only metals^12–14^, but some aluminium oxide ceramics (e.g. Al_2_O_3_), can be bonded using this method^15^. In particular, a typical bonding mechanism of Ag to Al_2_O_3_ was suggested; Ag ions temporarily generated during the redox reaction attach to the Al_2_O_3_ surface, leading to the formation of an initial Ag layer that facilitates the sintering of the AgNPs. Thus, an application of the bonding method to ceramics with high ion-binding properties was proposed. However, very little is known about the bonding morphology between the Ag and Si-materials as they have the high covalent properties, which requires surface pre-treatments even when using the AgNP joining process^4,16^.

Many studies have been conducted on the diffusivity of Ag into SiO_2_ and the adhesion between these materials^17–23^. The adhesion and diffusion of Ag into pure SiO_2_ is often difficult^17^, unlike some glasses containing sodium or potassium, e.g. soda-lime glass, into which Ag diffuses easily due to the ion exchange reaction^18,19^. The attraction of Ag ions to the SiO_2_ surface is enhanced after surface chemical treatments^20,21^, such as amination or the rupture of Si–O–Si bonds by ultrasonic radiation^22^ or electron irradiation^23^. It was also shown that the diffusion of metallic ions into SiO_2_ is facilitated under an electric field^24,25^. The diffusion of Ag ions into SiO2 without such an external driving force has not be studied in detail to our knowledge, but the formation of metallic ions would be likely to contribute to the bonding of SiO_2_ with metals.

During the reaction between Ag_2_O and an organic solvent, AgNPs can be produced by two different processes; a redox reaction temporarily producing Ag ions, and thermal decomposition during heating^26–28^, which can induce the formation of Ag ions or atoms as an intermediate state. A silicon oxide (i.e. SiO_x_) layer is generally formed on the surface of Si-materials as a native oxide film. We hypothesized that the decomposition of Ag_2_O could be used in the bonding of Ag to various Si-materials by taking advantage of the good adhesion between the intermediate forms of Ag and the silicon oxide. Here, we report the direct bonding of Si-materials, such as Si, SiO_2_, and SiC, by Ag sintering derived from the two different decomposition reactions of Ag_2_O. For the bonding of Si-materials, a thermal process was firstly designed based on the investigation of the decomposition behaviour of a paste comprising Ag_2_O and organic solvent using thermal analysis. Then, the influence of the bonding temperature on the joint strength was evaluated in addition to interfacial microstructural observations. Finally, the adhesion behaviour of the AgNP on the SiO_2_ substrate was modelled and compared to the experimental results.

## Results

### Evaluation of the bond quality of silicon-based materials

**Figure 1:**
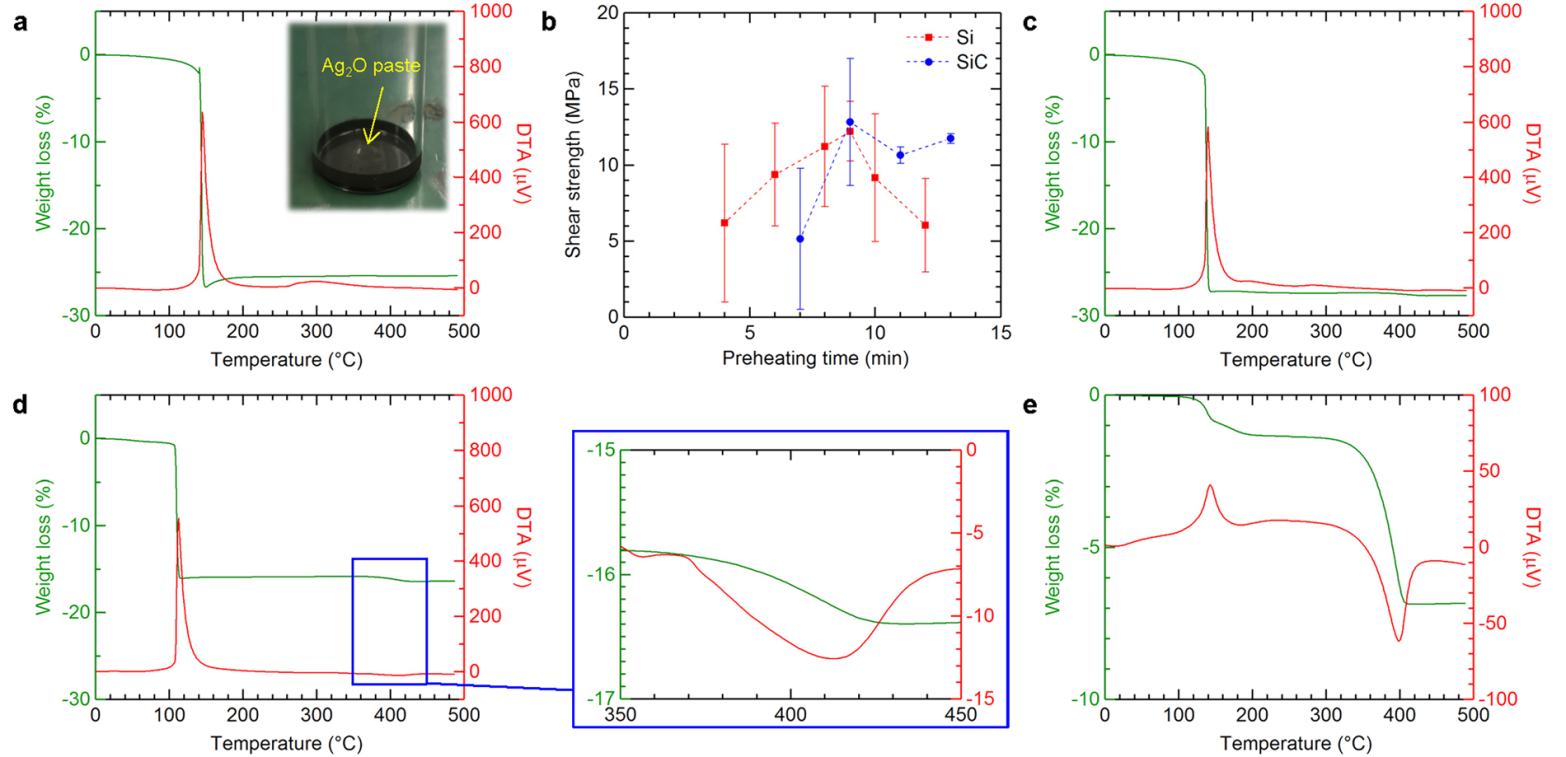
Weight loss and heat of reaction behaviour during heating to the bonding temperature. (**a**) TGA-DTA curves for the paste without preheating; the inset shows a photograph of the Ag_2_O paste used in this study. (**b**) Shear strength of the joint bonded at 400 °C after preheating at 100 °C for various holding times. (**c**–**e**) TGA-DTA curves for the paste after preheating for (**c**) 3 min, (**d**) 9 min, and (**e**) 13 min, where the positive/negative DTA values refer to exothermic/endothermic reactions, respectively.

To utilize the decomposition reaction of a paste comprising Ag_2_O and diethylene glycol (C_4_H_10_O_3_), control of the preheating before the bonding is useful in order to remove excess solvent, which is likely to hinder the sintering of particles. In addition, it is also necessary to limit the preheating temperature below the reduction temperature to avoid promoting this reaction, as the initial formation of excess amounts of AgNPs during the preheating process could result in their cohesion and inhibition of sintering. Figure 1(a) shows thermogravimetric analysis-differential thermal analysis (TGA-DTA) results of the Ag_2_O paste with increasing temperature. An exothermal DTA peak accompanied by significant weight loss in TGA was measured at 145 °C; it was reported that the redox reaction of Ag_2_O exhibits an exothermic reaction above 100 °C^13,14^. Subsequently, another exothermic DTA peak was also measured at 297 °C, attributed to the decomposition of the organic solvent around 300 °C. The optimum preheating temperature was therefore chosen as 100 °C. The preheating time appropriate for bonding was investigated based on an evaluation of the bond quality. Figure 1(b) shows the shear strength of joints between Si-materials (Si and SiC) and Au using the Ag layer bonding method at 400 °C plotted against preheating time over the range of 2–13 min. The shear strength of both joints increased upon reaching 9 min, above which it decreased again. In order to verify the behaviour before and after 9 min, TGA-DTA curves were measured as a function of temperature for samples preheated for 3, 9, or 13 min, as shown in Fig. 1(c–e), respectively. For the short preheating time of 3 min (Fig. 1(c)), a slight exothermic DTA peak was observed, indicating the presence of remaining solvent. An endothermic DTA peak accompanied by weight loss was observed from 350 to 400 °C for the moderate and long preheating times of 9 min and 13 min, respectively (Fig. 1(d,e)). Endothermic reactions generally occur during the thermal decomposition of Ag_2_O at around 400 °C^19,26^. It should be noted that the rate of weight loss during the reduction of Ag_2_O decreased with increasing preheating time. The results revealed that the amount of reduced Ag decreased because of excess volatilization of organic solvent during preheating. Hence, it is considered that remaining Ag_2_O particles that were not fully reduced by the organic solvent were thermally decomposed in the case of longer preheating times. Further, the slight thermal decomposition behaviour may have contributed to the bonding because the behaviour was also confirmed in appropriate preheating time for joint shear strength (Fig. 1(d)). Thus, the bonding process was performed with a preheating time of 9 min at 100 °C.

**Figure 2:**
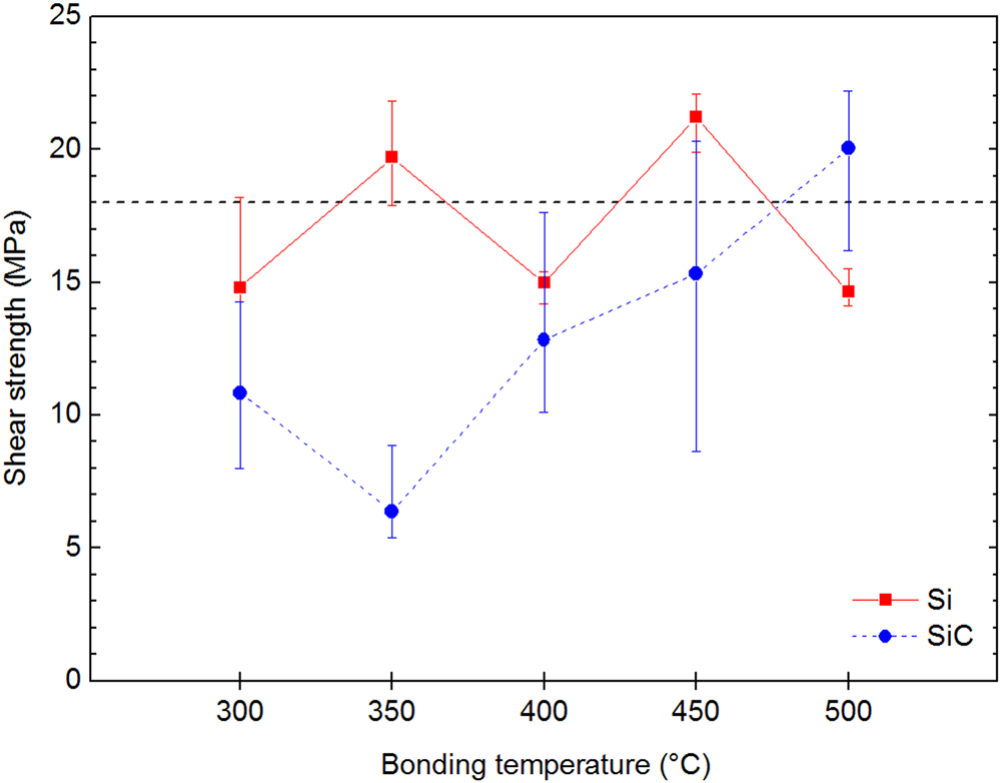
Shear strength of the joints between Si-materials and Au prepared via AgNP sintering. Si-Au and SiC-Au joints prepared at 300–500 °C were tested.

Figure 2 shows the shear strength of the joint between Au and Si-materials bonded by the sintering of Ag derived from the decomposition of Ag_2_O. It was found that the bonding temperature influenced the shear strength of the Si and SiC joints. Both joints showed maximum shear strengths comparable to that of Pb-5Sn solder (18–25 MPa)^29,30^. Hence, we achieved good bonding of Au to Si or SiC without any surface modification. On the other hand, a decrease in the strength occurred for both the Si and SiC joints around 350–400 °C. The fracture in the Si and SiC joints occurred in the sintered Ag layer for almost all bonding temperatures, except for 500 °C, where it occurred in the Si substrate in the Si joint and at the SiC/Ag interface for the SiC joint. Although SiO_2_/Ag bonding was also performed at 300–500 °C, the joint shear strength was poor regardless of the bonding temperature. This was because the fracture occurred in the SiO_2_ substrate, revealing that the SiO_2_/Ag interface was well bonded. The change in the fracture location with increasing bonding temperature indicates increased thermal stresses acting at the interface or substrate. The thermal stress results in the degradation of the joint strength caused by thermal shock, particularly for brittle materials such as Si and SiO_2_.

**Figure 3:**
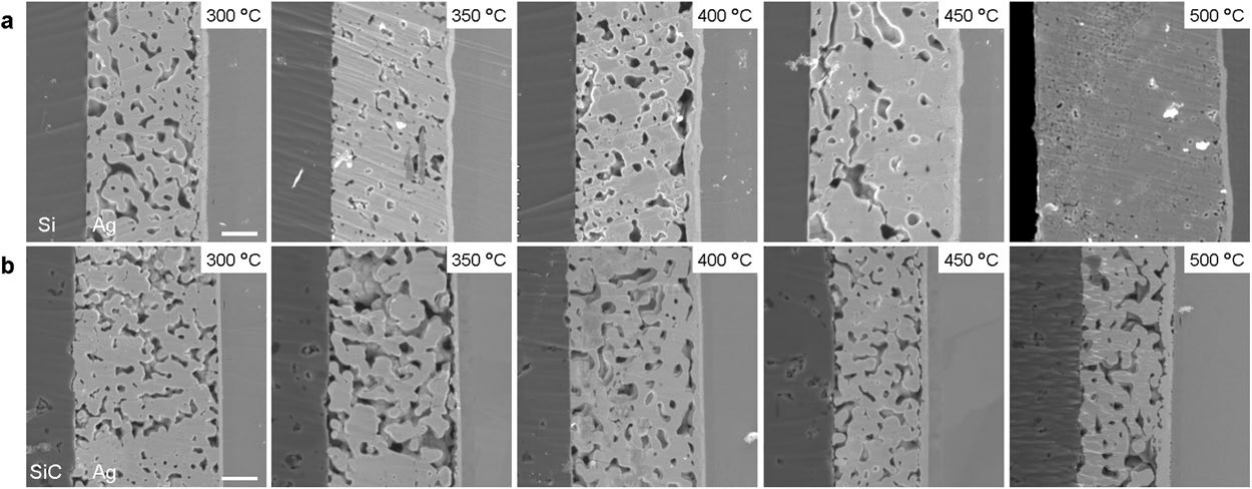
Cross-sectional microstructures of the joints between Si-materials and Au showing the porosity of the Ag layer as a function of temperature. (**a**) Si-Au and (**b**) SiC-Au joints at bonding temperature of 300, 350, 400, 450, and 500 °C. Scale bars: 5 μm. All images have the same magnification.

Figure 3 show cross-sectional SEM images of the Si and SiC joints bonded via the sintered Ag layer from 300 to 500 °C. The sintered Ag sufficiently adhered to the substrates with a few pores; Ag adequately bonded to Au, as observed in previous studies^7,15^. Depending on the bonding temperature, there were differences in the presence of pores in the Ag layer; the porosity tended to decrease with the increasing temperature. A large porosity was observed for samples prepared at 400 °C for the Si joint and 350 °C for the SiC joint, which are temperatures consistent with those where the shear strength decreased. Further, the fracture locations were almost all in the Ag layer, despite the presence of pores at the interface between the Ag and Si-materials. We assume that the porosity in the Ag layer directly influenced the joint shear strength and that the strength of the Si-materials/Ag interface was comparably higher than that of the Ag layer; the correlation between joint strength and porosity will not be satisfied in the case of low interfacial strength. Hence, direct bonding of Ag and Si-materials would be realized via the formation of a sufficiently strong interface for both Si-materials.

### Bonding morphology of Ag onto Si-materials

**Figure 4:**
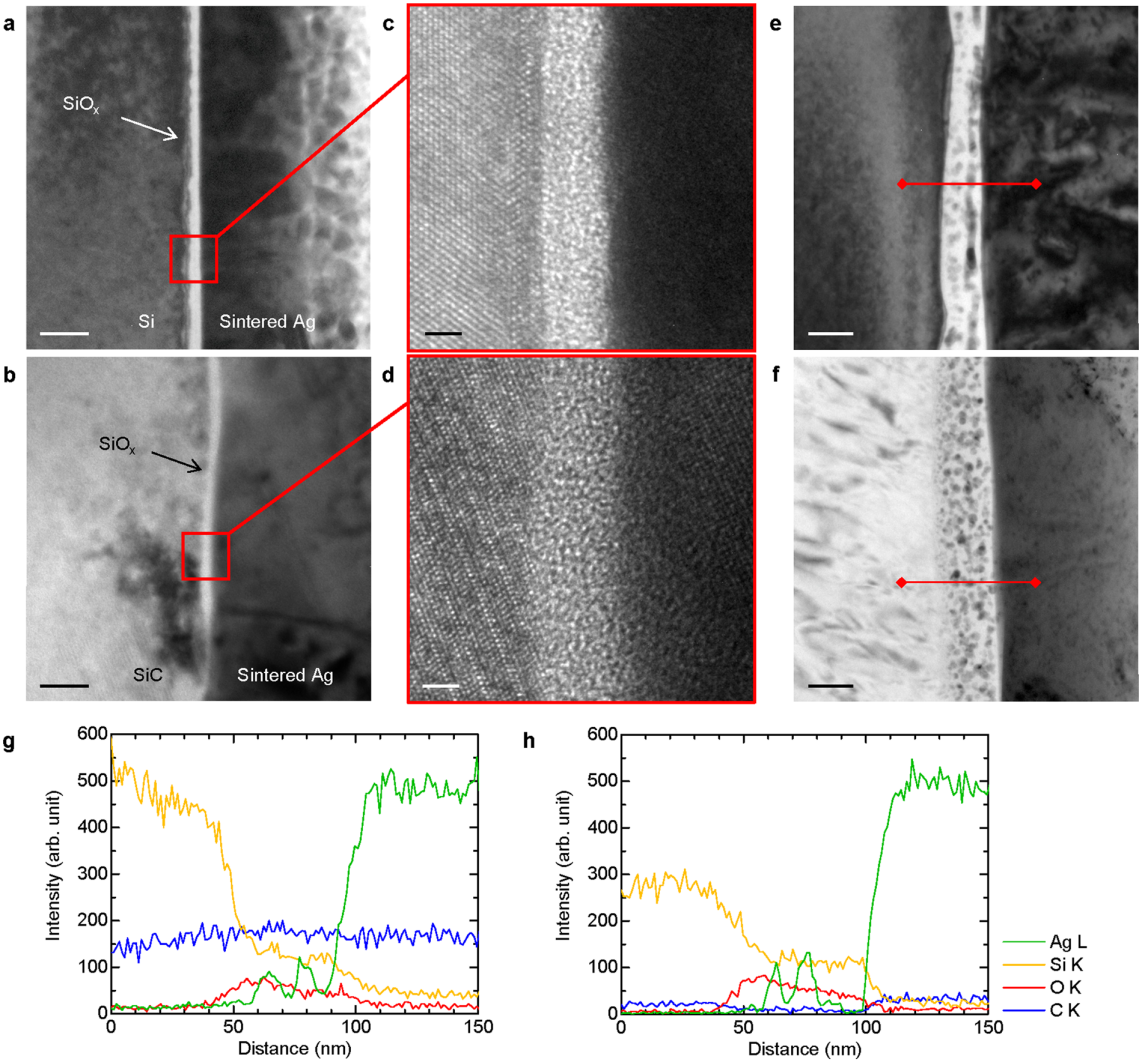
Microstructure and composition of the interface between Si-materials and Ag. Bright-field (BF) TEM image of the interface between (**a**) Si and Ag and (**b**) SiC and Ag bonded at 300 °C. (**c**,**d**) High-resolution TEM image of (**a**,**b**) showing the bonding of Ag and Si-materials via an amorphous native oxide. Interface between (**e**) Si and Ag and (**f**) SiC and Ag bonded at 500 °C. (**g**,**h**) EDS line analysis for Ag, Si, O, and C along the red line shown in (**e**,**f**), respectively. Scale bars, (**a**,**b**) 20 nm, (**c**,**d**) 2 nm, and (**e**,**f**) 50 nm.

Figure 4 shows TEM images of the microstructure of the interfaces between Si-materials and Ag obtained at 300 and 500 °C. The Si and SiC joints bonded at 300 °C (Fig. 4(a,b), respectively) showed the sintered Ag adhering well to the substrates. An intermediate layer with a thickness below 5 nm was observed at the interface between the Si-materials and sintered Ag (Fig. 4(c,d)). Since a native oxide usually covers Si-materials (e.g., Supplementary Fig. S1 showing the TEM image of the cross-section of the initial Si substrate before bonding), this layer was expected to correspond to the silicon oxide film. The results revealed that bonding of the Si-material and Ag was achieved via this native oxide layer at 300 °C, which is not surprising considering the high quality of the SiO_2_ joint. On the other hand, the interfacial microstructure of the Si and SiC joints bonded at 500 °C (Fig. 4(e,f), respectively) were distinctly different from those bonded at 300 °C. The sintered Ag densely bonded to a thicker intermediate layer (50 to 100 nm). In addition, fine particles were present in the intermediate layer for both joints, where the particle size decreased towards the silicon side (from 10 nm at the Ag side to 1 nm at the Si-material side).

EDS line analyses were performed along the red lines shown in Fig. 4 (e,f) in order to identify the elemental composition of the intermediate layer and the fine particles. Figure 4(g,h) show the elemental distributions at the interface of Si and SiC joints bonded at 500 °C, respectively, for Si, O, Ag, and C. The O intensity increased at the location of the intermediate layer and Si was also identified. In addition, the intensity of C did not change in the layer. These results reveal that the intermediate layer corresponds to silicon oxide rather than the organic solvent or voids. Both results showed an increase in Ag intensity corresponding to fine particles identified as AgNPs. Therefore, it was found that interfacial bonding between Ag and Si-materials at 500 °C was achieved via a silicon oxide layer containing AgNPs with sizes from several to 10 nm, which were thought to form between 300 °C and 500 °C.

**Figure 5:**
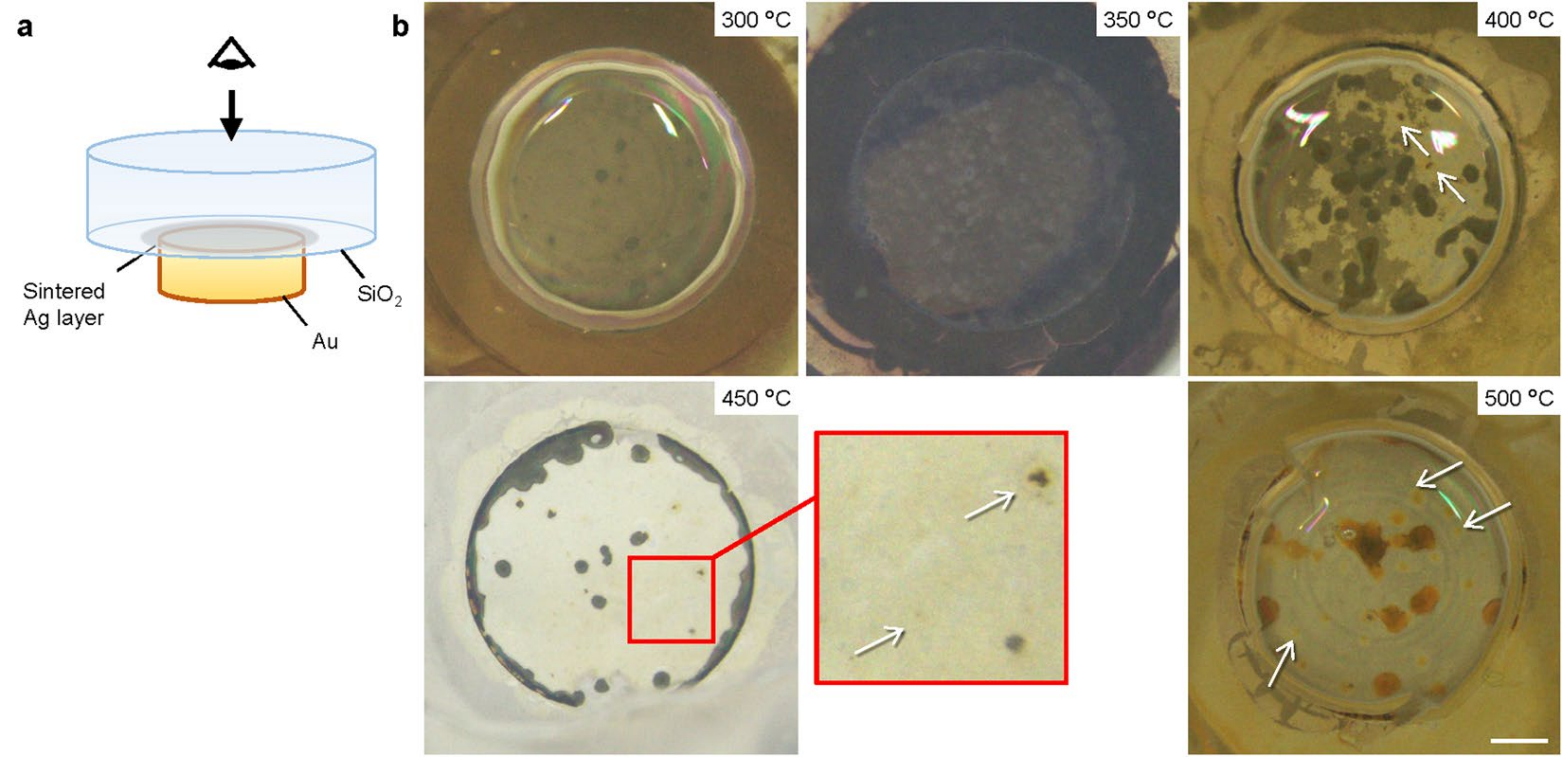
Stereomicroscopic images showing the visible colour change of SiO_2_ bonded to Au using Ag_2_O paste as a function of temperature. (**a**) Schematic diagram of the procedure for stereoscopic observation, where the diameter of the Au disc was 5 mm. (**b**) Top views of the interface at 300–500 °C for every 50 °C, where the arrows indicate areas of colour change. The white regions correspond to the sintered Ag layer. Scale bar: 1 mm.

The evolution of the interfacial structure was also observed as a visible colour change in the samples. Figure 5 shows stereomicroscopic images of SiO_2_-Au joints observed from the SiO_2_ side (Fig. 5(a)). Although there were no significant differences observed between the joints bonded up to 350 °C, a visible colour change occurred above 400 °C. A few yellow-coloured regions were observed at 400 °C, which increased in size with increasing temperature. Almost the entire bonded region exhibited either a yellow or brown colour at 500 °C. Such visible changes in SiO_2_ or silicate glass have been reported as the result of doping with Ag ions, clusters, or NPs (e.g. the use of ion exchange), which implied that the particle growth determined the visible colour change of the glass^19,31,32^. Particularly, Simo *et al*. reported that Ag ions embedded in a soda-lime silicate glass grew into Ag molecular clusters or NPs at a threshold temperature of 410 °C, accompanied by an obvious colour change^19^. Specifically, it was revealed that the change in visible colour corresponded to the growth of AgNPs contained in the SiO_2_ layer, where an increase in the thickness of the layer resulted in further colour changes.

The oxidation of Si or SiC does not occur significantly under atmospheric conditions below 500 °C. In contrast, thermal decomposition of Ag_2_O occurs around 400 °C (as shown in Fig. 1), which is evident as the colour begins to change. In general, Ag and oxygen were generated during the thermal decomposition of Ag_2_O as follows^33^:

1 $${{\rm{2Ag}}}_{2}{\rm{O}}({\rm{s}})\to \mathrm{4Ag}({\rm{s}})+{{\rm{O}}}_{{\rm{2}}}({\rm{g}})$$

However, the reaction should actually involve the temporal formation of O before the formation of O_2_, resulting in an increase in the oxygen activity. The value of the standard Gibbs free energy change for oxide formation for Si is more negative than that for Ag. Hence, Si can react with oxygen before the formation of gaseous oxygen as follows:

2 $${{\rm{2Ag}}}_{2}{\rm{O}}({\rm{s}})+{\rm{Si}}\to \mathrm{4Ag}({\rm{s}})+{{\rm{SiO}}}_{2}({\rm{s}})$$

Indeed, Fields reported that the reaction occurred in glass frit bonding^34^. When Si-materials are oxidized, the volume change ascribed to the reaction generally occurs following the Pilling-Bedworth ratio *R*_PB_ defined as:

3 $${R}_{PB}={V}_{{\rm{oxide}}}/{V}_{{\rm{metal}}}={M}_{{\rm{oxide}}}\cdot {\rho }_{{\rm{metal}}}/n\cdot {M}_{{\rm{metal}}}\cdot {\rho }_{{\rm{oxide}}}$$

where *V*_m_ and *V*_o_ are the volume of, *ρ*_m_ and *ρ*_o_ are the densities of the metal and oxide, respectively, *M*_o_ and *M*_m_ are the atomic mass numbers of the metal and oxide, respectively, and *n* is the number of metal atoms in the oxide molecule^35^. *R*_PB_ for Si/SiO_2_ is about 2, while that for SiC/SiO_2_ is nearly 1^36^, generally resulting in differences in the oxide layer roughness. In the present study, the oxide layer produced at the Ag/Si interface was rougher than that for the Ag/SiC sample, which indicates the remarkable oxidation of Si-materials during the thermal decomposition of Ag_2_O. It is considered that the formation of a silicon oxide layer containing AgNPs, as shown in Fig. 4(c,d), was induced through thermal decomposition of Ag_2_O. Conversely, the joints bonded at low temperature (Fig. 4(a,b)) did not undergo thermal decomposition, implying that Ag was predominantly formed by the redox reaction. Thus, the developed interfacial structure should change according to the thermal decomposition temperature. We note here that almost none of the fracture locations are in the Ag/silicon oxide interface. This suggests that the interfacial strength of Ag/silicon oxide exceeded that of the sintered Ag layer, and also that a strong interfacial bond was already established at 300 °C. Therefore, there is a possibility that the predominant mechanism resulting in the good interfacial strength is similar for both samples before and after thermal decomposition.

**Figure 6:**
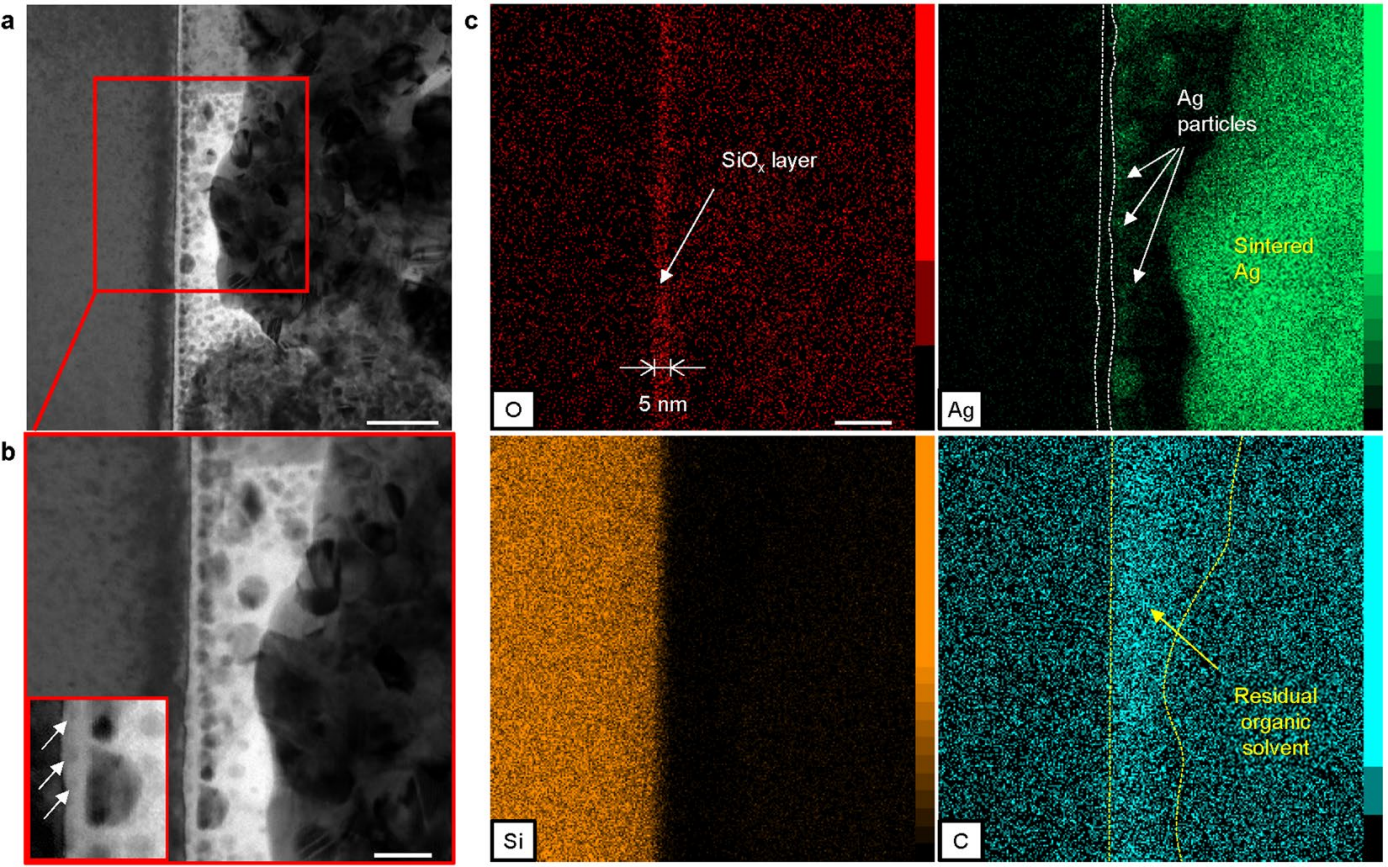
Adhesion of reduced AgNPs onto the native silicon oxide at the Si/Ag interface. (**a**) Bright-field TEM (BF-TEM) image showing the presence of nanoparticles in the thin layer. (**b**) Magnified BF-TEM image of (**a**) showing smaller nanoparticles present within the native oxide. (**c**) EDS mapping of Ag, Si, O, and C. Residual organic solvent hindered the adhesion of the sintered Ag to the silicon oxide. Scale bars: (**a**) 50 nm and (**b**,**c**) 20 nm.

Figure 6 shows TEM images of another region of the Ag/Si interface bonded at 300 °C, where the sintered Ag did not bond well to the Si (Fig. 6(a)). Fine particles were present in the white region between the sintered Ag and Si, which adhered well to the thin smooth layer (Fig. 6(b)). Figure 6(c) shows the corresponding EDS maps for O, Ag, Si, and C. It was found that fine particles near the layer were Ag. Unlike the joint bonded at 500 °C, the thickness of the high-O-concentration region was limited to 5 nm, nearly identical to that of the native silicon oxide observed in Fig. 4(a), whereas the concentration of C increased between the silicon oxide and sintered Ag layers. Hence, the white region was identified as residual organic solvent. Residual solvent usually hinders sintering of particles^37^; hence, this was identified as the reason for the poorly bonded interface. On the other hand, we assumed that the interfacial structure with insufficient sintering was equivalent to that developed from the intermediate state during bonding. These results imply that AgNPs could sufficiently adhere to the silicon oxide layer. It was also confirmed that some particles (~several nm) were present near the Si substrate inside the native oxide, as shown in Fig. 6(a), and that the sintered Ag layer penetrated the native oxide layer, as shown in the inset of Fig. 4(a).

## Discussion

Here we discuss the bonding process at each temperature range. Various processes are typically used to achieve AgNPs inside silicon oxide, e.g. using ion-implantation, the sol-gel method, or diffusion of Ag into silicon oxide by ion exchange, which require ions such as sodium^19^. However, here bonding was achieved at a low temperature via the native silicon oxide layer (comprising only silicon and oxygen), which reveals that such conventional formation processes cannot be available. On the other hand, the thin silicon oxide layer on the Si-material is likely to permit the diffusion of Ag ions, even at low temperatures (below 500 °C)^24,38^. Actually, Ag was observed near the interface between Si and native oxide as shown in Fig. 6(b). Further, it was reported that Ag ions could adhere to the surface of silicon oxide^21^. In the present study, during the reaction between Ag_2_O and the reducing solvent, silver carboxylate (R-COOAg) was temporarily formed, and subsequently Ag is thought to have precipitated as clusters or NPs along with the formation of gas, such as carbon dioxide and hydrogen oxide^39–41^. It should be noted that no oxygen was generated during the reaction, implying that the oxidation of Si-materials never occurs. This assumption supports our results that no growth of the silicon oxide layer was observed at 300 °C. It is thought that Ag ions first adhered to the silicon oxide and then, with increasing temperature, either diffused (in small quantities) or formed nanoparticles on the surface of the silicon oxide. AgNPs locally precipitated as the result of supersaturation of diffused Ag in the silicon oxide, which conversely indicates that Ag atoms or ions would be present inside the layer, particularly near the interface. Accordingly, the formation of the interfacial structure below the thermal decomposition temperature would be as follows: (I) the generation of Ag ions due to the reaction between Ag_2_O and organic solvent, (II) adhesion of Ag ions onto native oxide, (III) diffusion of Ag ions or atoms into the native oxide with increasing temperature, and (IV) precipitation of AgNPs followed by sintering. On the other hand, considering Si has a greater affinity for oxygen than Ag, the reaction between Ag_2_O and Si described above (Reaction (2)) could not be negligible also for the presence of Ag in the SiO_2_, i.e., corresponding to the step (III). Since the oxidation reaction of Si by Ag_2_O involves the production of Ag and SiO_2_, Ag could be confined by the produced SiO_2_ on the native oxide at atomic scale. Nonetheless, the confined Ag would obey the step (IV).

Considering the formation of the interfacial structure above the thermal decomposition temperature of Ag_2_O, we observed many AgNPs formed in the thick silicon oxide layer. As described above, it is widely known that the thermal decomposition of Ag_2_O can finally result in AgNPs^3,28,37^. Here we try to elucidate the formation of the silicon oxide layer containing AgNPs above 300–500 °C. Since no melting behaviour of silicon oxide occurred, the interfacial structure could be derived from several source: (i) confinement of generated Ag during the growth of silicon oxide, and (ii) thermal diffusion of Ag into silicon oxide. Concerning (i), Ag is confined by silicon oxide that is additionally formed on the surface of the native oxide as the result of reaction (2) described above; these reactions might occur repetitively. Since thermal decomposition occurs with a peak at around 400 °C, Ag should be present with a varying concentration distribution in the silicon oxide layer, corresponding to the observed experimental results. There are many reports regarding the oxidation mechanism in Si-materials; the oxidation mechanism changes with the thickness of the silicon oxide layer^42–44^. In the case of a thin silicon oxide layer (below 20–30 nm), Si atoms emitted from the substrate couple with oxygen near the surface of silicon oxide, which causes fast oxidation. In contrast, inward diffusion of oxygen becomes more dominant than silicon diffusion with the increase in the thickness, and the growth of silicon oxide occurs either inside the layer or at the interface of the Si-material/silicon oxide (Deal-Grove model)^42^. The model is generally applicable for the oxidation progress corresponding to a thickness >20–30 nm. Therefore, the growth of silicon oxide occurs not at the surface but either inside the layer or at the interface of the Si-material/silicon oxide. In the present study, the thickness of the interfacial layer was about 50 nm. Therefore, the above mechanism (i) would be possible as the initial mechanism for the formation of interfacial structure since the oxidation of Si by Ag_2_O at the surface of silicon oxide can occur up to 20–30 nm.

Considering mechanism (ii), some studies have reported the diffusion of Ag in silicon oxide despite the difficulties mentioned above. For instance, it was shown that Ag ions dissolved in glass, prepared by the mixture of glass frit with Ag paste, diffuses out^45–47^. Further, in the case of an ion implanted SiO_2_ film, it was observed that AgNPs precipitated in the SiO_2_ at a deeper level than the implanted range after annealing at 500 °C^48,49^. Actually, AgNPs were found inside the SiO_2_ at a distance of several tens of nanometres from the SiO_2_/Ag interface for the SiO_2_ joint (Supplementary Fig. S5). These results implied the possibility of thermal diffusion of Ag atoms or ions below 500 °C. Assuming the thermal diffusion of Ag, we can explain the observed size- and concentration-distribution of the AgNPs in the silicon oxide layer. The Ag and oxygen concentrations would be distributed according to the thermal decomposition rate of Ag_2_O dependent on the temperature, with a peak at about 400 °C. After the precipitation of AgNPs via the same precipitation mechanism below the thermal decomposition temperature, the size of AgNPs was in proportion to the concentrations, i.e., to the nearest-neighbour distances^50,51^.

As above, the formation of the interfacial structure could be attributed to both mechanisms. Specifically, Ag would be initially present in silicon oxide owing both to the thermal diffusion of Ag and the confinement by silicon oxide through the oxidation of Si by Ag_2_O. Subsequently, Ag would be present over the entire grown silicon oxide layer owing to the diffusion of Ag.

Such a formation process of the silicon oxide layer containing AgNPs is similar to that below the thermal decomposition temperature, where the presence of AgNPs is evidence of the existence of many Ag ions or atoms in the silicon oxide layer during bonding. Therefore, Ag atoms or ions inside the silicon oxide, particularly near the interface, should be available effectively for sufficient bonding between the silicon oxide and Ag in both cases.

**Figure 7:**
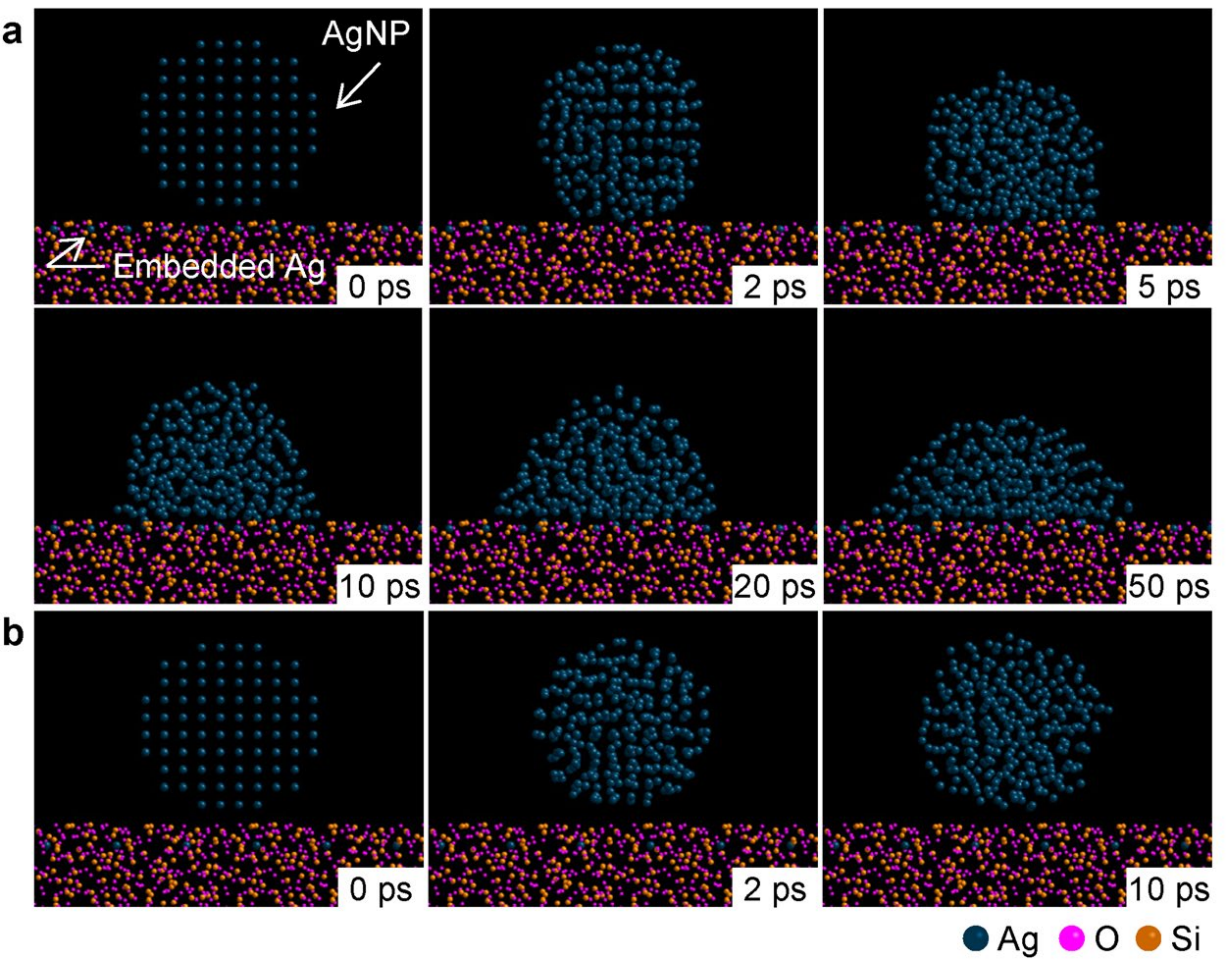
Adhesion behaviour of AgNP onto SiO_2_ dependent on the interfacial structure of SiO_2_, determined via molecular modelling. (**a**) Adhesion process of AgNP onto the SiO_2_ surface with an embedded atomic Ag layer at *d* = 1*r* from the surface. AgNP can adhere to SiO_2_ with increasing wetting angle owing to the presence of embedded Ag atoms. (**b**) Desorption process of AgNP from SiO_2_ with an embedded Ag layer at *d* = 3*r* from the surface, showing no attractive force between the AgNP and SiO_2_ layer. These simulations were performed at 500 °C.

It was indicated that the *in situ* formation of a silicon oxide layer containing Ag atoms contributes to the direct bonding of Ag and Si-materials in both temperature ranges. Herein, molecular dynamics simulations were used to elucidate the adhesion behaviour between Ag and SiO_2_. In order to verify the hypothesized mechanism, three kinds of amorphous SiO_2_ substrate were analysed, SiO_2_ substrates with Ag atoms embedded at *d* distance from the interface simulating the SiO_2_ containing atomic Ag in correspondence with Fig. 4; *d* values were *r*, 2*r*, and 3*r* where *r* is the Ag atomic radius. Figure 7 describe the adhesion of a AgNP onto the SiO_2_ with Ag atoms embedded at (a) *d* = *r* and (b) *d* = 3*r*. It was confirmed that a AgNP is not wet on the SiO_2_ with Ag atoms embedded at *d* = 3*r* at 500 °C, which reveals that there is no strong attractive force between Ag and SiO_2_ atoms. In contrast, we found that AgNP can adhere to the SiO_2_ with Ag atoms embedded at *d* = *r* and 2*r* and increase the wetting angle. In addition, the layer should also contribute to the adhesion, even at 300 °C. It was reported that precipitated Ag in the SiO_2_ layer remains in a metallic state^52^, indicating that Ag is present along the Ag/SiO_2_ interface and able to maintain the metallic bonding state. Therefore, it was revealed that the presence of Ag near SiO_2_ surface played a significant role in establishing the bonding between Ag and Si-materials in either temperature range. Furthermore, it is also suggested that the interlayer between Ag and Si-materials can be designed by the control of the oxidation of Si-materials using the thermal decomposition process of Ag_2_O paste.

Consequently, we found that the bonding between Ag and Si-materials was realized via *in-situ* formation of a silicon oxide layer containing AgNPs resulting from the different decomposition reactions of Ag_2_O: (i) redox reaction between Ag_2_O and the organic solvent, producing Ag ions and AgNPs, and (ii) thermal decomposition of Ag_2_O producing Ag ions, AgNPs, and also oxygen, inducing oxidation of the Si-materials at low temperature. The dominant decomposition reaction can be controlled by the initial Ag_2_O paste design or control of the thermal process. Thus, this method is expected to enable the implementation of various bonds with Si-materials; not only low-temperature direct bonding, but also direct fabrication of MOS structures and the production of conductive films by controlling the Ag concentration and SiO_2_ layer thickness. Furthermore, in addition to bonding processes, the decomposition reaction of Ag_2_O could be widely applied in various fields, such as the fabrication of conductive paths in various materials^28^, and development of the screen-printing process for solar cells^45,46^.

## Methods

### Paste Preparation and characterization

Commercial silver oxide (I) (Ag_2_O) particle diameters of 2–3 μm (Kojundo Chemical Laboratory Co. Ltd.) were used as the bonding material. The particles were milled for 10 min using an agate mortar and mixed with diethylene glycol (C_4_H_10_O_3_) as the organic agent at a concentration of 180 μl/g. The mixture was processed to form a Ag_2_O paste for bonding using a planetary centrifugal mixer (Thinky AR-100). The thermal characteristics of the Ag_2_O paste were measured by performing differential thermal analysis (DTA) and thermogravimetric analysis (TGA) (Rigaku TG8120) at a heating rate of 60 °C/min in air.

### Bonding Processing

Si wafers with a (100) orientation (Komatsu Silicon America, Inc.), polycrystalline silicon carbide (SiC) prepared by sintering (Kyocera Co., Ltd.), and amorphous silicon dioxide (SiO_2_) (Kojundo Chemical Laboratory Co., Ltd) were used as the Si-material substrates for bonding. The dimensions were 10 mm × 10 mm × 0.6 mm or Si, 10 mm × 10 mm × 1 mmt for SiC, and 10 mm in diameter × 5 mm for SiO_2_. Gold (Au) discs with 5 mm diameter and 2 mm thickness were used as the metal substrate, which was fabricated by nickel/gold plating with a thickness of 3 μm and 1 μm, respectively, on a copper disc. Ag_2_O paste was applied to the surface of the Si-material substrates with a thickness of 50 μm and then preheated at 100 °C. The Au substrate was placed on the Ag_2_O paste layer after preheating. The samples were heated to the bonding temperature of 300–500 °C at a rate of 60 °C/min in an infrared heating furnace under air condition and held for 5 min with a pressure of 5 MPa. The samples were cooled by forced air after bonding.

### Experimental Characterizations

In order to evaluate the influence of bonding temperature on the bond quality, the shear strengths of Au/Si-material joints bonded at 300–500 °C with a pressure of 5 MPa were measured with a strain rate of 30 mm/min. The shear strength was evaluated as the average value of three joints, and the error bars were determined as the standard deviation values. The joint microstructures were observed using a stereomicroscope (Olympus SZX7). The cross-sectioned joints were prepared by using a cross-section polisher (JEOL SM-09010) and observed using a scanning electron microscope (SEM; Hitachi S-3000H). The interfacial microstructure between the sintered Ag layer and the Si-materials was investigated using transmission electron microscopy (TEM; JEOL JEM-2100F) combined with energy dispersive X-ray spectroscopy (EDS). Thin foil samples for TEM observations were prepared using a focused ion beam method (FIB; JEOL JIB-4500).

### Molecular Dynamics simulations

Calculations of the adhesion behaviour of AgNP onto SiO_2_ were performed using the commercial molecular dynamics software SCIGRESS ME 2.0 (Fujitsu). For the potential functions, Generalized Embedded Atom potential (Ag–Ag), Morse potential (Si–O), and ME3Organic potential (Ag–N, Ag–O and Ag–Si), which are included in the MD software package, were used. The simulated temperature and pressure were set to 500 °C and 1 atm, respectively. The SiO_2_ substrate was designed as amorphous and Ag atoms were embedded at *d* = *r*, 2*r* and 3*r*, where *r* is the atomic radius of Ag, from the surface of the substrate. The diameter of the AgNP on SiO_2_ was 2 nm.

### Data availability

The datasets generated and analysed during the current study are available from the corresponding author upon reasonable request.

## Electronic supplementary material

Supplementary material
