# Supplementary material for: Silver oxide decomposition mediated direct bonding of silicon-based materials
Source: Sci Rep. 2018 Jul 11;8:10472. doi: 10.1038/s41598-018-28788-x (PMC6041264; doi:10.1038/s41598-018-28788-x)
Supplement: Supplementary file 1 — Supplementary material [file 41598_2018_28788_MOESM1_ESM.docx]

**Silver oxide decomposition mediated direct bonding of silicon-based materials**

**Tomoki Matsuda^1*^, Kota Inami^1^, Keita Motoyama^1^, Tomokazu Sano^1^ & Akio Hirose^1^**

^1^Division of Materials and Manufacturing Science, Graduate School of Engineering, Osaka University, 2-1 Yamadaoka, Suita, Osaka 565-0871, Japan.

*Corresponding author: t-matsu@mapse.eng.osaka-u.ac.jp


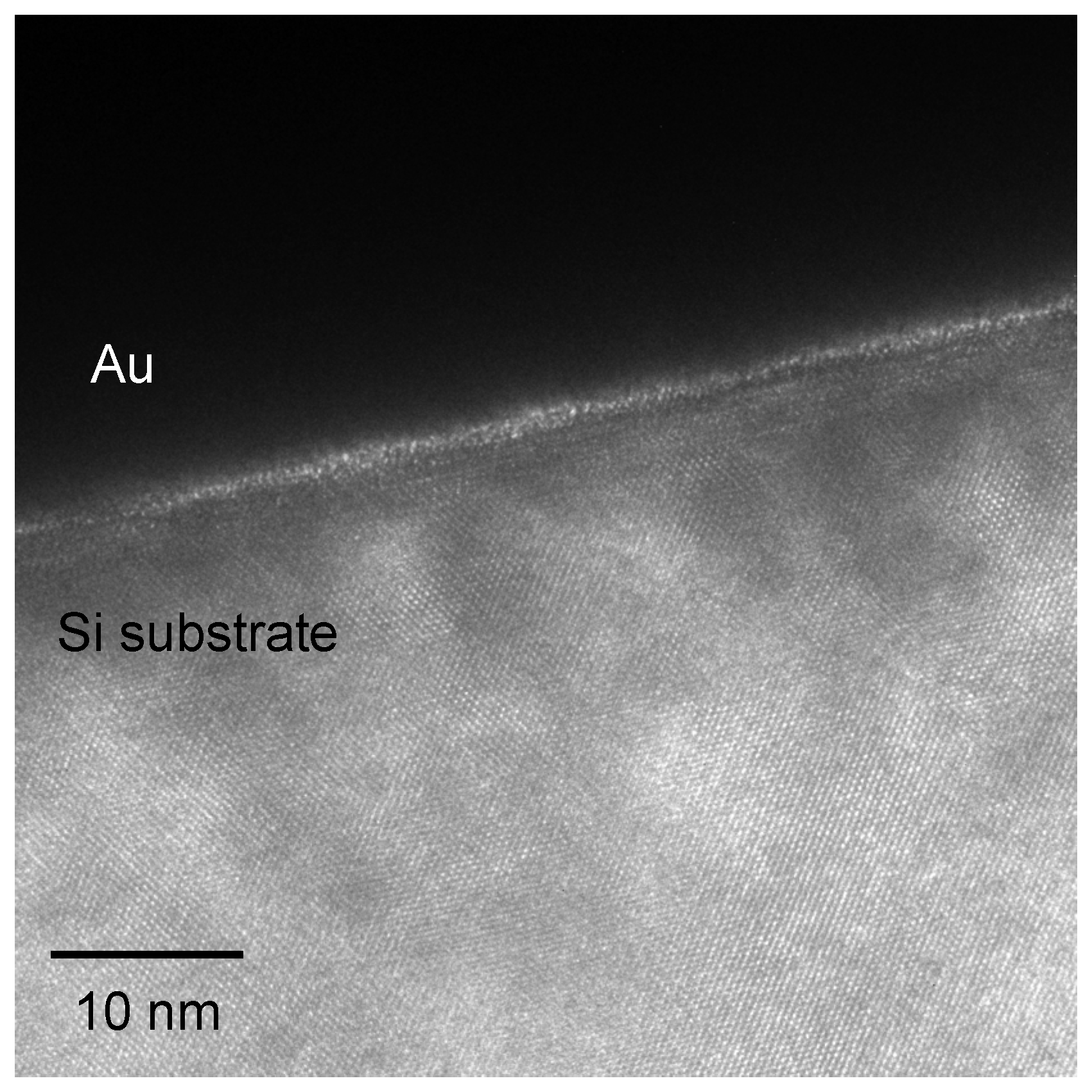


**Supplementary Figure S1 | Cross-sectional TEM image of Si substrate before bonding.** Native oxide layer is present on the Si surface. The black region corresponds to coating material (Au). For preparation of the TEM sample, the surface of the Si substrate was coated with gold using an E-1030 sputtering coater (Hitachi), and the TEM sample was picked up from the surface using focused ion beam processing.


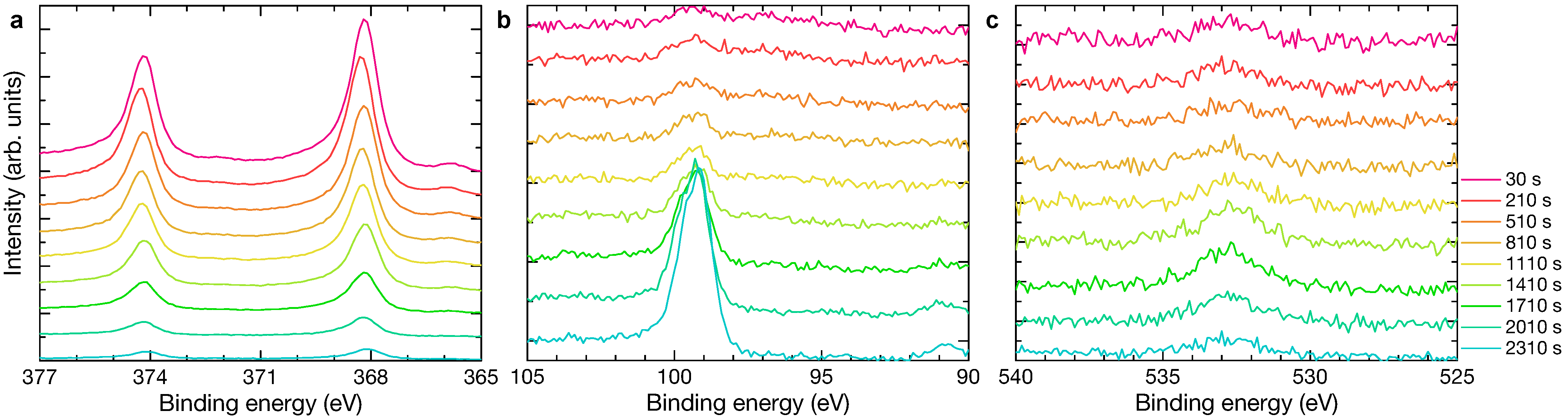


**Supplementary Figure S2 | Chemical bonding condition between Ag and Si substrate.** X-ray photoelectron spectroscopy profiles of the Ag/Si interface after argon sputtering from the Ag to the Si side: (a) Ag 3d, (b) Si 2p, and (c) O 1s. The experiment was performed on the fractured surface of the Ag/Si joint bonded at 500 °C. The intensity of Ag 3d peak decreased with the sputtering time, while that of the Si 2p peak increased, which showed the analysis direction from the mainly sintered silver layer to the Si substrate. The O1s peak appeared between 1110 s and 2010 s with the maximum intensity at 1710 s. Additionally, a different peak of Si 2p was confirmed at 103.7 eV after the sputtering for 1710 s. These results indicate that the silicon oxide layer was present exactly between Ag and the Si substrate.


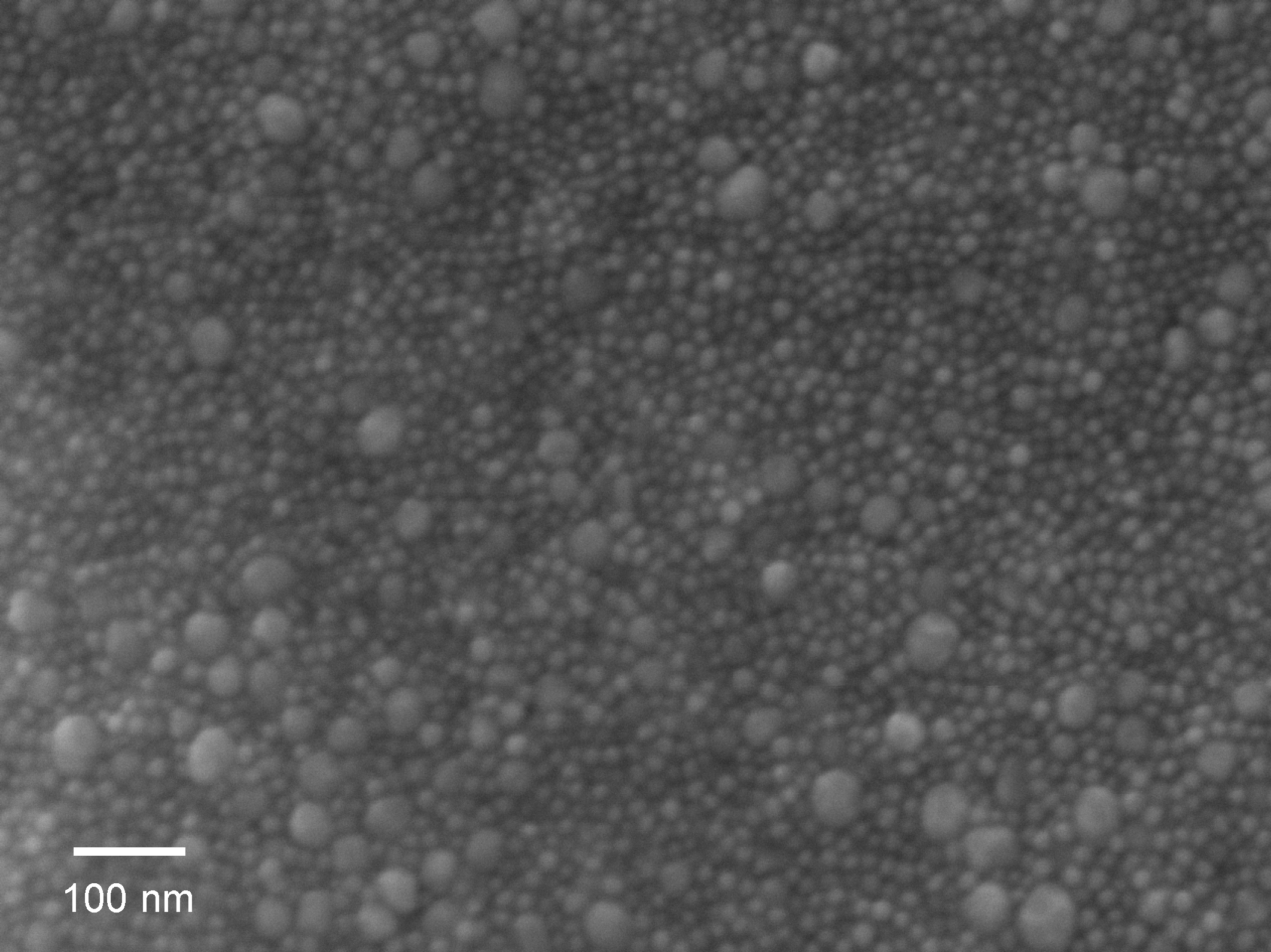


**Supplementary Figure S3 | FE-SEM image of the comparative Ag nanoparticles material.** Commercial Ag nanoparticles with particle sizes of 5–30 nm (low-temperature sintering silver ink) were used as comparative materials for comparison.


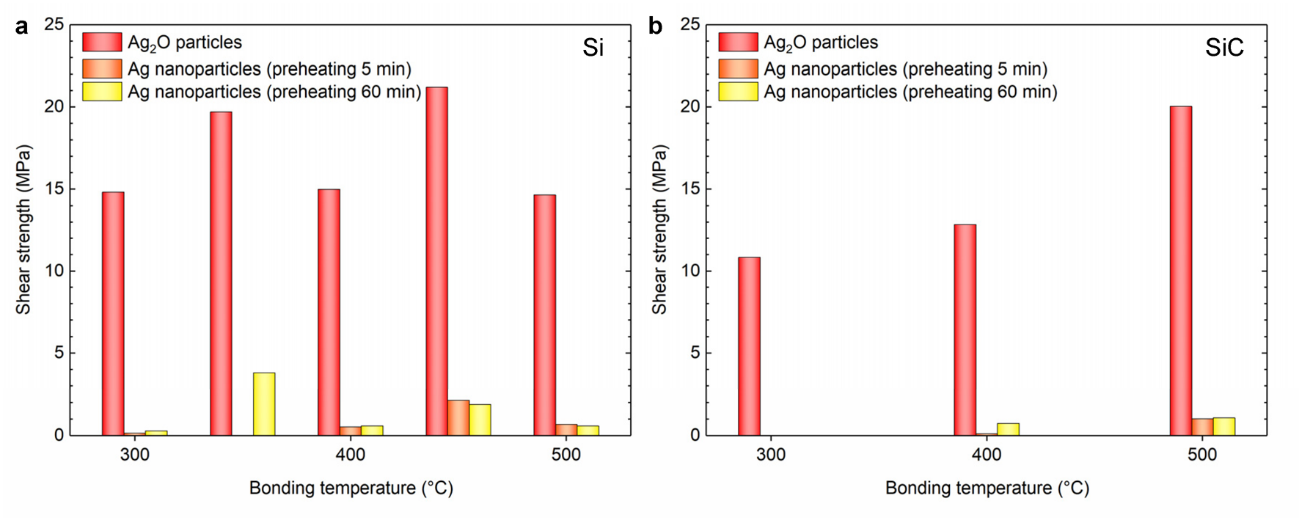


**Supplementary Figure S4 | Comparison of the bond quality of Si/Ag and SiC/Ag joint between Ag_2_O and Ag nanoparticles.** Bonding of (a) Si or (b) SiC and Ag using Ag nanoparticles was also performed for comparison. The samples were heated to 300–500 °C and held for 5 or 60 min after the preheating at 100 °C for 10 min. Unlike the Ag_2_O particles, the Ag nanoparticles hardly enabled the bonding in spite of the increase in the bonding temperature. This result indicated that the decomposition of Ag_2_O fundamentally contributes to the bonding of silicon-based materials.


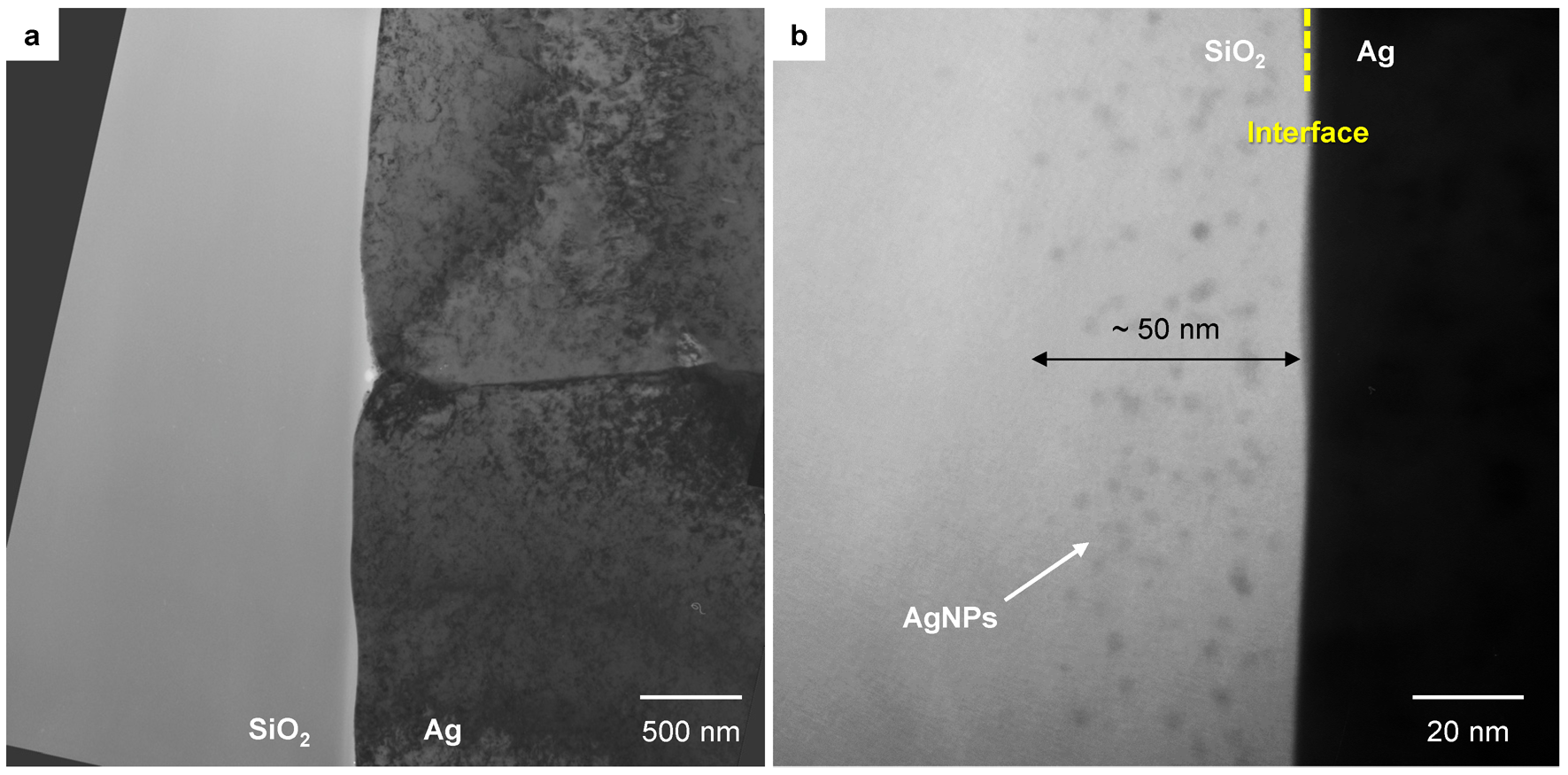


**Supplementary Figure S5 | Bright-field TEM images of the joint interface between high-purity amorphous SiO_2_ and sintered Ag layer bonded at 500 °C using the Ag_2_O paste.** Overview (a) and magnified view (b) of the joint interface. High-purity Synthetic Quartz glass (Shin-Etsu Quartz Products Co., Ltd.) was used as amorphous SiO_2_ substrate for a TEM experiment. Even for the SiO_2_/Ag joint, Ag particles with 1–5 nm diameters are present inside SiO_2_ at 50 nm from the SiO_2_/Ag interface. This result can reveal the diffusion of Ag into the silicon oxide layer regardless of the ion exchange reaction which is usually applied to glasses containing some additives.
